# Differential Diagnosis of *Entamoeba* spp. in Clinical Stool Samples Using SYBR Green Real-Time Polymerase Chain Reaction

**DOI:** 10.1155/2014/645084

**Published:** 2014-02-12

**Authors:** Thiago dos Santos Gomes, Mariana Coimbra Garcia, Flavia de Souza Cunha, Heloisa Werneck de Macedo, José Mauro Peralta, Regina Helena Saramago Peralta

**Affiliations:** ^1^Departamento de Doenças Infecciosas e Parasitárias, Faculdade de Medicina, Universidade Federal do Rio de Janeiro, 21941-902 Rio de Janeiro, RJ, Brazil; ^2^Instituto de Microbiologia Prof. Paulo de Góes, Universidade Federal do Rio de Janeiro, 21941-590 Rio de Janeiro, RJ, Brazil; ^3^Faculdade de Medicina, Universidade Federal Fluminense, 24030-210 Niterói, RJ, Brazil; ^4^Departamento de Patologia, Hospital Universitário Antônio Pedro, Rua Marquês do Paraná 303, Prédio Principal, 4° andar, Centro, 24030-210 Niterói, RJ, Brazil

## Abstract

Amoebiasis, a disease caused by *Entamoeba histolytica*, is usually diagnosed by microscopic examination, which does not differentiate the morphologically identical species of the *E. histolytica*/*E. dispar* complex. Furthermore, morphologically similar species such as *Entamoeba hartmanni* contribute to misidentification. Therefore, there is a need for more sensitive and specific methods. This study standardized a multiplex real-time PCR system for *E. histolytica* and *E. dispar* and a single real-time PCR for *E. hartmanni*. The multiplex protocol detected up to 0.0143 pg of *E. histolytica* DNA and 0.5156 pg of *E. dispar* DNA, and the average melting temperature (*T*_*m*_) was 73°C and 70°C, respectively. For *E. hartmanni*, the *T*_*m*_ was 73°C and the amplification was successful down to 0.03 fg of plasmid DNA. Negative controls and other intestinal parasites presented no amplification. Among the 48 samples tested, *E. dispar* DNA was detected in 37; none exhibited *E. histolytica* DNA and 11 were negative in the multiplex protocol. In 4 of these 11 samples, however, *E. hartmanni* DNA was amplified. SYBR Green is demonstrated to be an interesting option and these combined PCR reactions can improve laboratory diagnosis of amoebiasis in developing countries.

## 1. Introduction

The genus *Entamoeba* contains many species, six of which are found in the human intestinal tract: *Entamoeba histolytica*, *Entamoeba dispar*, *Entamoeba moshkovskii*, *Entamoeba coli*, *Entamoeba hartmanni*, and *Entamoeba polecki*. Of these species, only *E. histolytica* is associated with pathological injuries; the others are considered to be nonpathogenic species [1]. *E. histolytica* is responsible for approximately 50 million cases of amoebiasis worldwide each year, resulting in up to 100,000 deaths [2, 3]. Because of these characteristics, *E. histolytica* is globally considered to be a leading parasitic cause of human mortality [1]. However, historical epidemiological data concerning *E. histolytica* infections can lead to overestimates because they were obtained before the formal separation of this parasite into two morphologically identical species: the potentially pathogenic *E. histolytica* and the nonpathogenic *E. dispar* [4]. More importantly, it is also estimated that 90% of diagnosed infections are caused by *E. dispar* [5].

Diagnosis of *E. histolytica* has historically relied on microscopic examination of protozoan morphology. Optical microscopy is a very useful tool for the diagnosis of amoebiasis because it is simple and cheap to execute. However, concentration techniques and stained smears are required to increase its low sensitivity [1, 6]. It is also influenced by the intermittent release of cysts, requiring the examination of multiple samples from different days [7]. Moreover, this method cannot differentiate between morphologically identical species such as *E. dispar* and *E. moshkovskii*, and the proficiency of the examiner can also be crucial for precise identification, as other *Entamoeba* species can present structures very similar to the features of *E. histolytica* [2, 8, 9].

*Entamoeba hartmanni* is one of these species with similar morphological forms because its cysts share characteristics with those of the *E. histolytica*/*E. dispar*/*E. moshkovskii* complex. The major difference between these species is the size of the cysts, which in *E. hartmanni* are generally smaller than in those from the complex [4, 10]. For this differentiation to be conducted by microscopy, an appropriate microscope capable of performing morphometric analysis is also required. Given that, this organism can still be difficult to identify depending on the circumstances and the condition of the sample. Contractile trophozoites or cysts of *E. histolytica* can be erroneously identified as *E. hartmanni*, and larger structures of *E. hartmanni* can also be misdiagnosed as the pathogenic species. Alternative molecular methods could be helpful to provide accurate and reliable identification of *Entamoeba* species. Polymerase Chain Reaction (PCR), including real-time PCR, has provided the means to identify *E. histolytica*, *E. dispar,* and *E. moshkovskii* in a variety of samples [11–17]. However, little attention has been devoted to the possibility of identifying *E. hartmanni*, whose structures confuse examiners at different proficiency levels [8]. Previously, data determined by microscopic examination have demonstrated a prevalence of *E. hartmanni* of 13.3% in HIV patients and 2.5% in children in Brazilian groups [18, 19]. Identification of *Entamoeba* species other than the complex using molecular techniques has been conducted in only a single study in Brazil, in which the evaluation of Brazilian clinical samples previously identified as positive for the complex by microscopic examination demonstrated the presence of *E. hartmanni* DNA in more than one sample, while *E. moshkovskii* DNA was not detected among the samples analyzed [20]. These results showed the presence of *E. hartmanni* in Brazil but yielded no data about* E. moshkovskii*.

Real-time PCR is a very sensitive methodology that could become an important tool to achieve these goals, and SYBR Green (N′,N′-dimethyl-N-[4-[(E)-(3-methyl-1,3-benzothiazol-2-ylidene)methyl]-1-phenylquinolin-1-ium-2-yl]-N-propylpropane-1,3-diamine) technology with its probe-free assays is a less expensive choice for establishing more efficient protocols in developing countries [12]. Because of these possibilities, the objectives of this study were to standardize SYBR Green real-time PCR protocols for the identification of *E. histolytica*, *E. dispar,* and *E. hartmanni* and to evaluate their efficiency on clinical stool samples.

## 2. Material and Methods

### 2.1. DNA Samples

The positive controls used in the SYBR Green multiplex real-time PCR were genomic DNA from the standard strain of *E. histolytica* HM1-IMSS and *E. dispar* P2 (a strain isolated from patient samples in Piauí, Brazil). The DNA was measured in a Qubit fluorometer (Invitrogen, Carlsbad, CA, USA), and 2-fold dilutions in water were prepared for testing using the SYBR Green real-time PCR protocol. For *E. hartmanni*, however, a cloning procedure was necessary to obtain a positive control because a protozoan culture of *E. hartmanni* was not available. For this process, positive DNA samples characterized in a previous study were used [20]. The specific *E. hartmanni* sequence was amplified with primers EhartF and EhartR2; this fragment was then cloned using the pCR 2.1-TOPO vector as described in the protocol with the pCR 2.1-TOPO TA Cloning Kit (Invitrogen). The extraction and purification of plasmid DNA containing the cloned fragment were performed using the Illustra PlasmidPrep Mini Spin Kit (GE Healthcare UK Limited, Buckinghamshire, UK), according to the manufacturer's instructions. This cloned plasmid DNA was measured in a Qubit fluorometer (Invitrogen) and 10-fold dilutions in water were prepared for testing with the SYBR Green real-time PCR protocol. In both protocols, DNA solutions from other intestinal parasites (two of each) were tested for a specificity assessment: *E. moshkovskii*, *Cryptosporidium hominis*, *Giardia lamblia*, and *Schistosoma mansoni*.

### 2.2. Clinical Samples

Stool samples from 2263 patients who were treated at Hospital Universitário Antônio Pedro (HUAP), Niterói, Rio de Janeiro, Brazil, between August 2008 and June 2009 were submitted to a microscopic examination in order to detect the presence of parasites. The procedure was conducted at the HUAP's parasitology laboratory. Moreover, a group of 292 stool samples from individual residents of Sumidouro, Rio de Janeiro, Brazil, a rural area, was also subjected to the same examination. Those samples identified as positive for the *E. histolytica*/*E. dispar* complex were selected to evaluate this SYBR Green real-time PCR system. Additional stool samples from 50 individuals with negative direct parasitological examinations were included as negative controls. This study was reviewed and approved by the Human Investigation Committee of the Universidade Federal Fluminense with protocol number 020/07.

### 2.3. Microscopic Examination

The microscopic examination was conducted by HUAP's parasitology laboratory staff. Prior to morphologic analysis, this service concentrated the stools using a spontaneous sedimentation method [21]. Then the sediments were analyzed in wet mounts stained with Lugol's iodine solution. For the microscopic examination, the laboratory staff used a Nikon Eclipse E20 optical microscope (Nikon). After the analysis, the sediments were stored at −20°C for DNA extraction.

### 2.4. DNA Extraction

DNA templates were extracted using the Fast Prep DNA Kit and the FastPrep FP120 Disrupter (MP Biomedicals, Solon, OH, USA). Further Purification was performed using the QIAquick PCR purification Kit (QIAGEN Inc., Valencia, CA, USA), and the purified DNA was stored at −20°C. Both methods were performed according to the manufacturer's instructions.

### 2.5. Conventional PCR Amplification

A multiplex PCR reaction was performed according to a protocol previously described by Santos et al. [22], with some modifications. The specific primers for *E. histolytica* and *E. dispar* were previously defined by Núñez et al. [23], *E. histolytica* (EHP1—5′CGATTTTCCCAGTAGAAATTA3′ and EHP2—5′CAAAATGGTCGTCTAGGC3′) and *E. dispar* (EDP1—5′ATGGTGAGGTTGTAGCAGAGA3′ and EDP2—5′CGATATTGACCTAGTACT3′). These primer sets were used to amplify specific sequences of 132 bp for *E. histolytica* and 96 bp for *E. dispar*. Each reaction was performed in a volume of 50 *μ*L using PCR Supermix (Invitrogen), 20 pmol of each primer, 0.1% bovine serum albumin (BSA Sigma Chem. Co., USA), and 2 *μ*L of the DNA sample. The PCR reaction was carried out using a Veriti 96 Well Thermal Cycler (Applied Biosystems, CA, USA), and the amplification parameters were as follows: 3 minutes at 94°C; 30 cycles of 30 seconds at 94°C, 30 seconds at 55°C, and 30 seconds at 72°C; and finally 7 minutes at 72°C. The amplified product was visualized with ethidium bromide staining after electrophoresis on a 2.0% agarose gel.

### 2.6. Multiplex SYBR Green Real-Time PCR Standardization

This PCR reaction was standardized for the simultaneous detection and identification of *E. histolytica* and *E. dispar*. The specific primers used for the detection of *E. histolytica* and *E. dispar* were the same oligonucleotide sequences employed for conventional multiplex PCR. Initially, a single format was designed in order to amplify *E. histolytica* and *E. dispar* separately. This format allowed us to know the specific melting temperature (*T*_*m*_) of the sequence amplified from each species and the feasibility of standardizing a multiplex reaction with these primers. The simplex PCR cycling parameters consisted of 2 minutes at 50°C, 2 minutes at 95°C and 35 cycles of 15 seconds at 95°C and 33 seconds at 55°C, using Platinum SYBR Green qPCR SuperMix-UDG (Invitrogen) and the same PCR reagents and conditions. A dissociation stage was then performed; the parameters consisted of 15 seconds at 95°C, 1 minute at 60°C, 15 seconds at 95°C, and 15 seconds at 60°C. Finally, the multiplex format was standardized in a final volume of 25 *μ*L. Four different amounts of primers were tested: 1.25, 2.5, 3.75, and 5 pmol. Two amounts of ROX Reference Dye (Invitrogen) were also tested in the standardization process: 0.25 pmol and 1.25 pmol. All reactions were carried out in an ABI 7500 System thermocycler (Applied Biosystems) and the PCR cycling parameters were the same as described for the single format, with an additional comparison between two annealing temperatures: 55°C and 60°C. The amplified products were visualized with ethidium bromide staining after electrophoresis on a 2.0% agarose gel for confirmation.

### 2.7. Standardization of SYBR Green Real-Time PCR for Identification of *E. hartmanni*

DNA sequence alignment was performed using CLC Sequence Viewer software (CLC bio). The sequences aligned were rRNA genes of the highly homologous species *E. histolytica*, *E. dispar*, *E. moshkovskii,* and *E. hartmanni*. Specific regions in the *E. hartmanni* sequence (GenBank access: AF149907) were identified. A forward primer EhartF (5′-CGTTCAAGACATGAGTGTGA-3′) and two reverse primers (EhartR1—5′-CCGTAGATCTCCTATTCACTTT-3′); EhartR2 (5′-ACAACACATTCATGTCGCA-3′) were designed.

To verify the identity of the target sequence of the primers, a conventional PCR was performed in a volume of 50 *μ*L, using PCR Supermix (Invitrogen), 20 pmol of each primer, 0.1% bovine serum albumin (BSA Sigma Chem. Co), and 2 *μ*L of DNA sample. The PCR reaction was carried out using a Veriti 96-Well Thermal Cycler (Applied Biosystems), and the amplification parameters were as follows: 3 minutes at 94°C; 40 cycles of 30 seconds at 94°C, 30 seconds at 55°C, and 30 seconds at 72°C; and finally 7 minutes at 72°C. The amplified product was visualized by ethidium bromide staining after electrophoresis on a 2.0% agarose gel and sent to the sequencing platform of the Genomic Unit at the Laboratory of Cell Physiology Darcy Fontoura (Federal University of Rio de Janeiro, Brazil). After confirming the identity of the amplified product on the GenBank database, the standardization process of the real-time PCR began. The PCR reaction using primers EhartF and EhartR2 was performed in a reaction volume of 25 *μ*L, using Platinum SYBR Green qPCR SuperMix-UDG (Invitrogen). Analyses concerning the amounts of primers and ROX Reference Dye were conducted just as described in the previous section for the multiplex protocol. Also, the reactions were carried out in the same thermocycler. The PCR cycling parameters consisted of 2 minutes at 50°C, 2 minutes at 95°C and 35 cycles of 15 seconds at 95°C and 33 seconds at 60°C. A dissociation stage was then performed; the parameters consisted of 15 seconds at 95°C, 1 minute at 60°C, 15 seconds at 95°C, and 15 seconds at 60°C. The amplified products were visualized with ethidium bromide staining after electrophoresis on a 2.0% agarose gel for confirmation.

### 2.8. Stool Sample Evaluation

For a preliminary evaluation of the real-time PCR protocols during the standardization process, a selection of *Entamoeba* positive samples was run. Thus, only samples exhibiting structures of the *E. histolytica*/*E. dispar* complex under microscopic examination were analyzed by multiplex conventional PCR and multiplex real-time PCR for the accurate identification of *E. histolytica* and *E. dispar*. Those samples with negative results obtained by both methods were submitted for further investigation with real-time PCR for the identification of *E. hartmanni*.

## 3. Results

### 3.1. Multiplex SYBR Green Real-Time PCR

**Figure 1:**
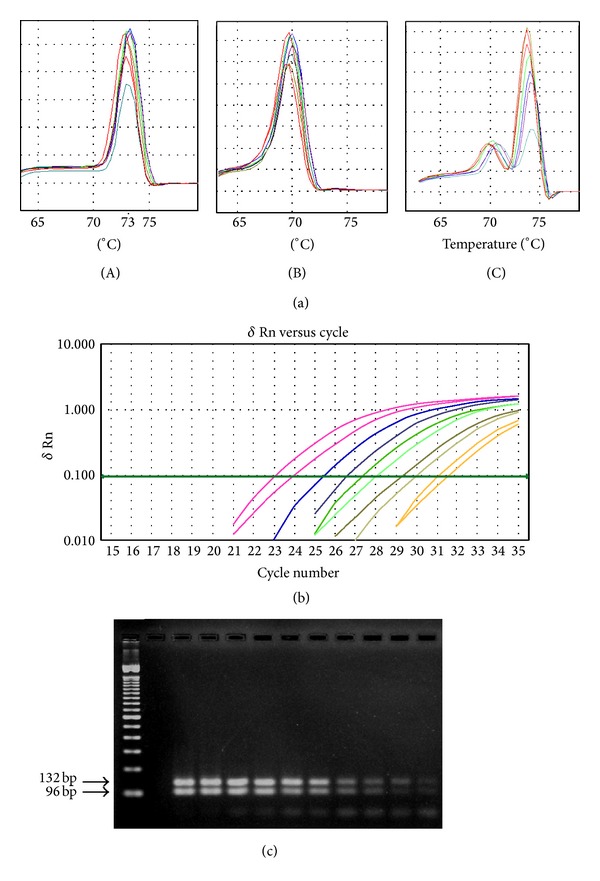
(a) Melting curve graph obtained through the SYBR Green real-time PCR for *E. histolytica* (A) and *E. dispar* (B) and the multiplex SYBR Green real-time PCR (C). (b) The software's graph demonstrating the DNA amplification of *E. histolytica* and *E. dispar* mixed DNAs in different amounts at the multiplex SYBR Green real-time PCR in descending order: 183.5 and 198.0 pg (*E. histolytica* and *E. dispar*, resp.); 91.5 and 99.0 pg; 45.9 and 49.5 pg; 22.9 and 24.7 pg; 11.5 and 12.4 pg; 8, 5.7 and 6.2 pg; 2.9 and 3.1 pg; 1.4 and 1.6 pg; 0.7 and 0.8 pg; 0.35 and 0.39 pg. (c) Agarose gel from multiplex real-time PCR products. Lane 1, 100 bp ladder standard; lane 2, no sample; lanes 3 to 12, PCR products in descending order of concentration.

The preliminary step represented by standardizing a single format of this reaction was essential to achieve the final protocol for the multiplex SYBR Green real-time PCR. This reaction determined that the mean melting temperatures (*T*_*m*_) of the amplified sequences of *E. histolytica* and *E. dispar* were 73.2°C (72.8 to 73.5°C) and 70.2°C (69.1°C to 70.9°C), respectively. These data demonstrated the possibility of standardizing a multiplex protocol to detect and identify these species using SYBR Green technology. Thus, multiplex real-time PCR using those primers was performed, and DNA amplification was exhibited as expected. The melting curve analysis demonstrated similar *T*_*m*_ values for each species, and the concomitant presence of genetic material from both species did not alter these conditions (Figure 1(a)).

Under the conditions for real-time PCR, the optimal concentration of primers was found to be 2.5 pmol. Other concentrations decreased the sensitivity of the assay or increased primer dimer formation. A concentration of ROX Reference Dye equivalent to 1.25 pmol gave the most reproducible and stable results. The best annealing temperature for amplification of both fragments was 55°C.

In the specificity assessment, no amplification was detected either in the presence of other intestinal parasite DNAs or in the negative control group. Specific DNA amplification was observed at all concentrations tested for both species, separately. For *E. histolytica*, amplification was tested successfully from 7.34 pg (*C*_*t*_: 21.2) to 0.0143 pg (*C*_*t*_: 30.2). For *E. dispar*, amplification was tested successfully from 264 pg (*C*_*t*_: 23.4) to 0.5156 pg (*C*_*t*_: 33.0). In order to model a mixed infection, genetic material from both species was tested together. A concomitant specific DNA amplification was observed at all concentrations tested, and the DNA of both species was amplified at levels as low as 0.3584 pg for *E. histolytica* and 0.3867 pg for *E. dispar* (Figures 1(b) and 1(c), Table 1). As expected, decreasing the DNA concentration also increased the *C*_*t*_ value, revealing an inverse relationship between these values. In the latter situation, the specific *T*_*m*_ value must be checked in the melting temperature graph to evaluate the presence of two peaks (Figure 1(a)).

### 3.2. SYBR Green Real-Time PCR for Identification of *E. hartmanni*

**Figure 2:**
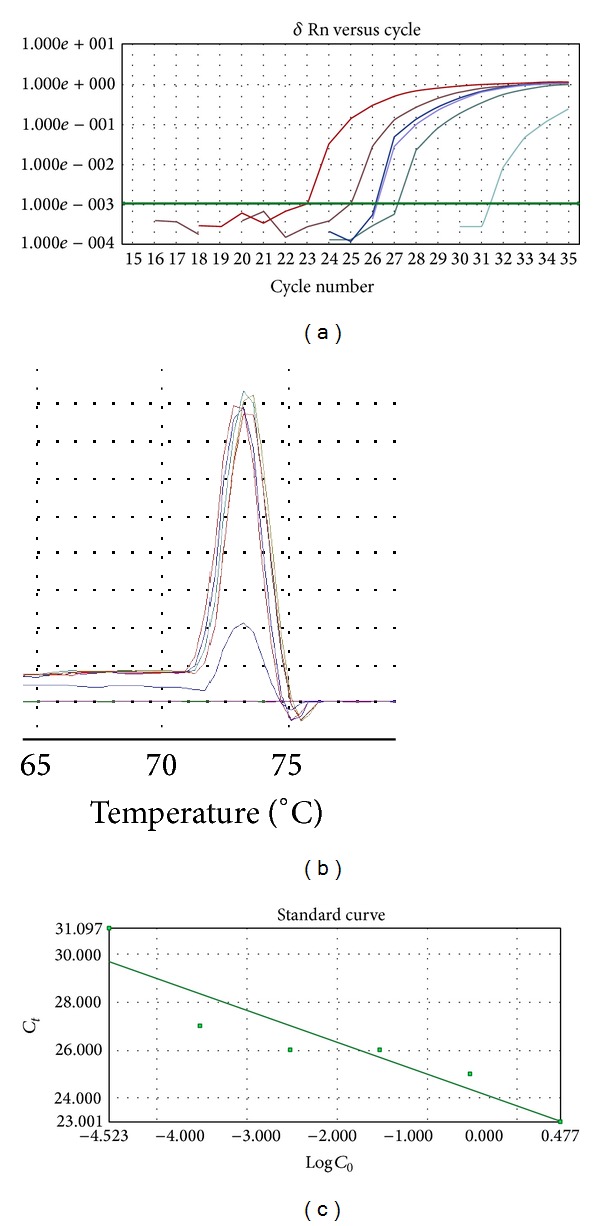
DNA amplification and the melting curve analysis using different DNA concentrations on SYBR Green real-time PCR for *E. hartmanni*. DNA concentrations: 3.0 pg, 0.3 pg, 0.03 pg, 0.003 pg, 0.0003 pg, and 0.00003 pg.

The alignment revealed highly specific regions in the *E. hartmanni* sequence that allowed the design of a forward primer (EhartF1) and a reverse primer (EhartR1). The fragment defined by these primers was 184 bp. However, GenBank database analysis showed a small region of homology with *Cryptosporidium hominis*. In an attempt to avoid homology with other intestinal parasites, a second reverse primer, EhartR2, was designed to exclude this homologous region. The new set of primers amplifies a sequence of 90 bp. In this real-time PCR protocol, the melting curve analysis revealed a mean melting temperature (*T*_*m*_) of 73°C for the amplified product (Figure 2). Agarose gel electrophoresis analysis confirmed the amplification of the specific 90 bp fragment. In the conditions for real-time PCR, the optimal concentration of primers was found to be 3.75 pmol. Other concentrations either decreased the sensitivity of the assay or increased primer dimer formation. For the ROX Reference Dye, 0.25 pmol was determined to be the concentration that gave the most reproducible and stable results.

In the specificity assessment, no amplification was detected either in the presence of other intestinal parasite DNAs or in the negative control group. Specific DNA amplification was observed down to a concentration of 0.03 fg. As noted above, the decrease in DNA concentration corresponded to an increase in the *C*_*t*_ value. The *T*_*m*_ value was between 72.8 and 73.6°C (Figure 2, Table 2).

### 3.3. Stool Sample Evaluation

**Figure 3:**
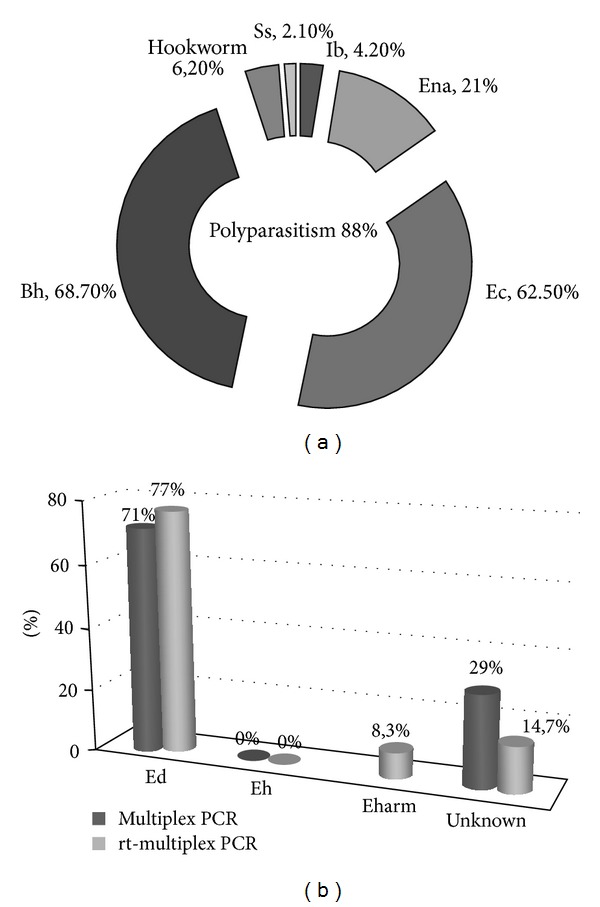
Results of the 48 samples with *Entamoeba histolytica/Entamoeba dispar* complex. (a) Frequency of polyparasitism and each detected parasite on microscopic examination. (b) Frequency of *E. histolytica* and *E. dispar* in conventional and multiplex real-time PCR and frequency of *E. hartmanni* by real-time PCR in negative samples from multiplex real-time PCR.

For this study, all samples were analyzed through microscopic examination. Of the 292 samples collected at Sumidouro, 24 specimens exhibited morphological structures characteristic of the *E. histolytica*/*E. dispar* complex, for a prevalence of 8.2%. In the HUAP's laboratory, these structures were detected in 24 specimens from the 2263 samples collected with a prevalence of 1.1%. These 48 samples were then selected and submitted to real-time multiplex PCR. The samples were also submitted to the corresponding multiplex conventional PCR format for comparison. Of these samples, 88% had polyparasitism with one or more parasites, mainly with *Blastocystis hominis* and *Entamoeba coli* (Figure 3(a)). The conventional multiplex PCR revealed amplification of the specific sequence of *E. dispar* in 34 samples, while real-time multiplex PCR was able to detect *E. dispar* in these 34 samples and in three other samples (77%). Amplification of the specific sequence of *E. histolytica*, however, was not observed in the samples tested by either methods (Figure 3(b)). The 11 samples negative for *E. histolytica* and *E. dispar* by both methods were then submitted to the SYBR Green real-time PCR protocol for *E. hartmanni* identification to investigate whether the morphological structure observed was misidentified. In four samples, the specific sequence of *E. hartmanni* was amplified while the remaining 7 samples remained unidentified (Figure 3(b)). To investigate the presence of inhibitory substances, these samples were contaminated with 11.7 fg of *E. hartmanni* DNA and subsequently submitted to *E. hartmanni* real-time PCR. DNA amplification was successful after this step, indicating that inhibitory substances did not influence the negative results.

## 4. Discussion

Several authors have described the crucial importance of accurate differentiation of *E*. *histolytica* from *E*. *dispar*, *E*. *moshkovskii*, and other morphologically similar species of *Entamoeba* for proper clinical management of patients and for assessment of their actual prevalence in different geographical regions [13]. To date, several microscopy-based epidemiological surveys to study the prevalence of *E*. *histolytica* and *E*. *dispar* have been performed in different parts of Brazil, but these studies were carried out without using molecular methods to accurately identify the species. In Brazil, the standard clinical approach is to treat all asymptomatic individuals who present with cysts in their feces with an antiprotozoal agent. This approach to treatment results in the indiscriminate use of antiamoebic drugs and has led to increased minimum inhibitory concentrations of these therapeutic agents against *E. histolytica*, with the potential for resistant strains to appear [24, 25].

In this study, we described the development of a real-time PCR system for the differential diagnosis of three species of *Entamoeba* that share identical or similar morphology as both cysts and trophozoites. The standardization of these PCR reactions is an important step in developing an efficient system for the identification of *Entamoeba* species. Most molecular diagnostic tools detect only *E. histolytica* and do not detect other morphologically identical or similar species, giving rise to several questions. A previous study has already demonstrated a situation in which samples that were positive by microscopic examination for the *E. histolytica*/*E. dispar* complex were identified as *E. hartmanni* after analysis by two different molecular methods [20]. To avoid this potential confusion and provide complete epidemiologic data on parasitic infections, it is important to develop a reliable system for the identification and differentiation of pathogenic and nonpathogenic parasites. For this approach, real-time PCR methodology is an interesting possibility because of its high sensitivity and specificity as well as its speed in processing samples, while SYBR Green technology represents a less expensive option for developing real-time PCR protocols in developing countries. Additionally, the SYBR Green approach is especially attractive for those who already have the conventional PCR assay running and want to convert to the real-time format [12].

The presented assays successfully amplified positive controls from *E. histolytica*, *E. dispar*, and *E. hartmanni*, demonstrating no amplification of DNA from other intestinal parasites. Furthermore, the amplification was successfully tested on amounts of DNA ranging between 7.34 pg and 0.0143 pg for *E. histolytica* and between 264 pg and 0.5156 pg for *E. dispar*. These results demonstrated a high sensitivity. Other studies have presented detection limits at higher DNA concentrations, such as 0.2 pg for *E. histolytica* and 2.0 pg for *E. dispar* [16, 26]. For *E. hartmanni*, amplification was observed even with a lower DNA concentration. However, the lack of *E. hartmanni* parasites must be considered in analyzing the *E. hartmanni* detection protocol, a limitation that was circumvented by subjecting the specific fragment amplified to a cloning procedure. Certainly, the high number of copies of the target sequence in the cloned plasmids allowed the amplification of specific *E. hartmanni* DNA with lower quantities of DNA.

Among the 48 samples that were diagnosed as containing the complex by microscopic examination, 37 samples were identified as *E. dispar*. However, there was no DNA amplification of *E. histolytica* among these samples, neither with conventional PCR nor with real-time PCR. These results demonstrate the importance of accurate identification of *Entamoeba* species by alternative and sensitive molecular methods, considering the relevance of these results to ensuring adequate treatment. Two samples with *E. dispar* also presented *Strongyloides stercoralis* and hookworms, parasites that need specific treatment. Because *E. histolytica* was not detected by 2 different and sensitive protocols, there is a high chance no antiamoebic treatment was actually necessary. These sample results can be compared to the natural distribution of the parasite in Brazil, which has remained unknown since *E. histolytica* was separated into two species. It is possible that the pathogenic species has a low prevalence in Rio de Janeiro, in southeast Brazil, while *E. dispar* exists at a higher frequency in this area. This hypothesis corroborates other studies conducted over the past few years in different parts of Brazil, which have suggested that *E. histolytica* is more common in the north and extreme northeast of Brazil and is rare in other regions [7, 27]. If this is true, accurate diagnosis becomes even more important to clinical conduct because other *Entamoeba* species can lead to misidentification.

The presence of other species identified as part of the complex is a problem that has been previously reported in the literature [22]. For this reason, this study also proposes the standardization of real-time PCR protocols for the identification of *Entamoeba* species other than the complex. Of the samples analyzed, 11 samples remained unidentified after the multiplex real-time PCR protocol and were submitted to screening for *E. hartmanni* DNA; this species' DNA was successfully amplified in four samples. Seven further samples remained negative after this analysis. However, further microscopic examination of these samples revealed another possible explanation for this negative result: the presence of *Entamoeba coli* cysts in the majority of these samples. It is possible that the presence of immature cysts in those samples resulted in a false positive diagnosis of the *E. histolytica*/*E. dispar* complex on microscopic examination.

Our results highlight the importance of accurate identification of *Entamoeba* species. In the diagnosis of amoebiasis, a simple detection at the level of the *E. histolytica/E. dispar* complex has been considered sufficient cause for clinical treatment. It is understandable for hospitals operating with limited funds to proceed in this manner. However, reference centers such as university hospitals or public health centers should use techniques such as PCR or antigen detection, described in the literature, for complete identification at the species level, of bacteria, viruses, fungi, and parasites [22, 28]. Accurate identification is extremely important for exact diagnosis and for providing correct epidemiological data.

Accurate diagnosis of amoebiasis is crucial in order for physicians to prescribe proper treatment. These laboratory results present important information that will help clinicians decide whether to apply an antiamoebic treatment or search for a different etiology for the presented symptoms. Only the specific detection of *E. histolytica* confirms a diagnosis of amoebiasis, while the identification of other nonpathogenic amoebas can lead clinical investigators to search for different pathologies with similar symptoms that would not be considered without this information.

## 5. Conclusion

The specificity and sensitivity demonstrated by the SYBR Green real-time PCR protocols indicate an interesting approach to the identification and differentiation of these *Entamoeba* species. The application of those protocols in different regions can be a helpful tool to provide more reliable parasitological diagnosis and more detailed epidemiological data on the prevalence of *Entamoeba* species around the world. The combination of both real-time PCR protocols may allow the development of a simple and useful *Entamoeba* identification system to improve the laboratory diagnosis of amoebiasis in developing countries.

## Figures and Tables

**Table 1:** Results of multiplex PCR obtained with different DNA concentration.

DNA (pg)		*C*_*t*_ value
*E. histolytica *	*E. dispar *	
183.5	198.0	23.1
91.5	99.0	23.9
45.9	49.5	25.4
22.9	24.7	26.5
11.5	12.4	27.3
5.7	6.2	28.1
2.9	3.1	29.3
1.4	1.6	30.0
0.7	0.8	31.1
0.36	0.39	31.5

**Table 2:** *C*_*t*_ and *T*_*m*_ values obtained with different DNA concentration from *E. hartmanni*.

DNA (pg)	*C*_*t*_ value	*T*_*m*_ (°C)
3.0	23.0	72.8
0.3	25.0	73.2
0.03	26.0	73.2
0.003	26.0	73.6
0.0003	27.0	73.2
0.00003	31.1	73.2
